# 

**Published:** 1902-09

**Authors:** 


### BOOKS RECEIVED.

International Clinics. A Quarterly of Illustrated Clinical Lectures and especially prepared Articles on Medicine, Neurology, Surgery, Therapeutics, Obstetrics, Pediatrics, Pathology, Dermatology, Diseases of the Eye, Ear, Nose and Throat, and other Topics of Interest to Students and Practitioners by leading Members of the Medical Profession throughout the World. Edited by Henry W. Cattell, A. M., M. D., Philadelphia, U. S. A., with the Collaboration of John B. Murphy, M. D., Chicago; Alexander D. Blackader, M. D., Montreal; H. C. Wood, M. D., Philadelphia; T. M. Rotch, M. D., Boston; E. Landolt, M. D., Paris; Thomas G. Morton, M. D., Philadelphia; James J. Walsh, M. D., New York; J. W. Ballantyne, M. D., Edinburgh, and John Harold, M. D., London, with Regular Correspondents in Montreal, London, Paris, Leipsic and Vienna. Volume II. Twelfth series. Philadelphia and London: J. B. Lippincott Company. 1902. [Cloth, $2.00 each.]

A Text-Book of Practical Therapeutics. With especial Reference to the Application of Remedial Measures to Disease and their Employment upon a Rational Basis. By Hobart Amory Hare, M. D., Professor of Therapeutics and Materia Medica in the Jefferson Medical College of Philadelphia. With special chapters by Drs. G. E. deSchweinitz, Edward Martin and Barton C. Hirst. New (9th) edition. In one octavo volume of 851 pages, with 105 engravings and 4 colored plates. Philadelphia and New York: Lea Brothers & Company. [Cloth, $4.00; leather, $5.00; half morocco, $5.50 *net*]

Woolsey's Surgical Anatomy. Applied Surgical Anatomy Regionally Presented, for the use of Students and Practitioners of Medicine. By Geo. Woolsey, A. B., M. D., Professor of Anatomy and Clinical Surgery in the Cornell University Medical College; Surgeon to Bellevue Hospital, etc. Octavo, 511 pages with 125 illustrations, including 59 full-page inset plates in black and colors. Philadelphia and New York: Lea Brothers & Company. 1902. [Cloth, $5.00 *net*; leather, $6.00 *net.*]

Grayson's Laryngology. A Treatise on the Diseases of the Throat, Nose and the Associated Affections of the Ear. By Charles P. Grayson, M. D., Lecturer and Instructor in Laryngology in the Medical Department, University of Pennsylvania. In one octavo volume of 540 pages with 129 engravings and 8 colored plates. Philadelphia and New York: Lea Brothers & Company. 1902 [Cloth, $3.00 *net.*]

The Theory and Practice of Infant Feeding. With Notes on Development. By Henry Dwight Chapin, A. M., M. D., Professor of Diseases of Children at the New York Post-Graduate Medical School and Hospital, etc. Octavo, pp. 335. Illustrated. New York: William Wood & Company. 1902. [Price, $2.25 *net.*]

The Medical Student's Manual of Chemistry. By R. A. Witthaus, A. M., M. D., Professor of Chemistry, Physics and Toxicology in Cornell University Medical College, New York City. Fifth edition. Octavo, pp. 689. New York: William Wood & Company. 1902. [Price, $3.50 *net.*]

A Physician's Practical Gynecology. By W. O. Henry, M. D., Professor of Gynecology in the Creighton Medical College, Omaha, Neb. First edition; 226 pages, 5 full-page illustrations and 61 illustrations in the text. Lincoln, Neb.: The Review Press. 1902. [Price, cloth, $2.00.]

The Principles and Practice of Bandaging. By Gwilym G. Davis, M. D., Assistant Professor of Applied Anatomy, University of Pennsylvania 12mo, pp. 157. Illustrated from Original Drawings by the Author. Philadelphia: P. Blakiston's Son & Company. 1902. [Price, $1.50 *net.*]

The Practical Medicine Series of Year Books. Comprising ten volumes on the year's Progress in Medicine and Surgery. Issued monthly under the general editorial charge of Gustavus P. Head, M. D., Professor of Laryngology and Rhinology in the Chicago Post-Graduate Medical School. Volume VIII. Pediatrics and Orthopedic Surgery. Edited by W. S. Christopher, M. D., John Ridlon, A. M., M. D., Samuel J. Walker, A. B., M. D., 1902. Chicago: The Year Book Publishers. [Price, $1.25; entire series, $7.50.]

Massage and the Original Swedish Movements. Their Application to Various Diseases of the Body. Lectures before the Training Schools for Nurses connected with the Hospital of the University of Pennsylvania, German Hospital and others of Philadelphia. By Kurre W. Ostrom, from the Royal University of Upsala, Sweden. Fifth edition, revised and enlarged; 115 illustrations. Philaadelphia: P. Blakiston's Son & Company. 1902. [Price, $1.00 *net.*]

Gibson & Russell's Physical Diagnosis. Third edition, revised and rewritten by Francis D. Boyd, C. M. G., M. D., F. R. C. P. E., Assistant Physician, Edinburgh Royal Infirmary. 12mo., pp. 465. With 144 illustrations. New York: D. Appleton & Company. Edinburgh and London: Young J. Pentland. 1902.

